# Safety, Tolerability, Pharmacokinetics, and Drug Interaction Potential of SPR741, an Intravenous Potentiator, after Single and Multiple Ascending Doses and When Combined with β-Lactam Antibiotics in Healthy Subjects

**DOI:** 10.1128/AAC.00892-19

**Published:** 2019-08-23

**Authors:** Paul B. Eckburg, Troy Lister, Susannah Walpole, Tim Keutzer, Luke Utley, John Tomayko, Ellen Kopp, Nicholas Farinola, Scott Coleman

**Affiliations:** a Spero Therapeutics, Cambridge, Massachusetts, USA; b Pfizer, Inc., New York, New York, USA; c E. Sullivan and Associates, LLC, Newburyport, Massachusetts, USA; d CMAX Clinical Research and Department of Clinical Pharmacology, Royal Adelaide Hospital, Adelaide, Australia; e Acceleron Pharma, Cambridge, Massachusetts, USA

**Keywords:** SPR741, drug interaction, pharmacokinetics, potentiator

## Abstract

SPR741 is a novel polymyxin B derivative, with minimal intrinsic antibacterial activity and reduced nonclinical nephrotoxicity compared to levels with polymyxin B, that interacts with the outer membrane of Gram-negative bacteria, enhancing penetration of coadministered antibiotics. The safety, tolerability, and pharmacokinetics (PK) of SPR741 were evaluated in two studies, after single and multiple intravenous (i.v.) doses in healthy adult subjects and after coadministration with partner antibiotics. In the single and multiple ascending-dose study, SPR741 or placebo was administered as a 1-h infusion at single doses of 5 to 800 mg and in multiple doses of 50 to 600 mg every 8 h (q8h) for 14 days. In the drug-drug interaction study, a single 400-mg i.v. dose of SPR741 was administered alone and in combination with piperacillin-tazobactam, ceftazidime, and aztreonam. PK parameters for SPR741 and partner antibiotics were determined using noncompartmental analysis. After single doses, a dose-linear and proportional increase in mean maximum concentration in plasma (*C*_max_) and area under the concentration-time curve (AUC) was observed. At doses of 100 to 800 mg, >50% of the dose was excreted in the urine in the first 4 h postdose. After multiple doses, the mean half-life was 2.2 h on day 1 and up to 14.0 h on day 14, with no evidence of accumulation after 14 days of dosing up to 400 mg. The PK profile of SPR741 and partner antibiotics was unchanged with coadministration. SPR741 was generally well tolerated at doses up to 1,800 mg/day. These data support further clinical development of SPR741 for treating serious infections due to resistant bacteria. (These studies have been registered at ClinicalTrials.gov under identifiers NCT03022175 and NCT03376529.)

## Abstract (precis)

SPR741 is a novel polymyxin B derivative, with minimal intrinsic antibacterial activity and reduced nonclinical nephrotoxicity compared to levels with polymyxin B, that interacts with the outer membrane of Gram-negative bacteria, enhancing penetration of coadministered antibiotics.

## INTRODUCTION

Rapid emergence of multidrug-resistant (MDR) Gram-negative bacteria is increasingly recognized as a cause of increased morbidity and mortality worldwide (1, 2). The rise in the incidence of infections caused by MDR bacteria poses a significant problem for patients with serious, potentially life-threatening infections because of the lack of available and effective treatment options (3–6). Thus, a critical need exists for new treatments that are effective against MDR bacteria (7).

SPR741 is a fully synthetic polymyxin analog that exhibits synergy in combination with coadministered antibiotics against many Gram-negative bacteria, including MDR pathogens (8–10). SPR741 lacks significant antibacterial activity as a stand-alone agent but interacts with the outer membrane of Gram-negative bacteria to increase permeability and thereby improve the accumulation of coadministered antibiotics inside the pathogen (8, 9). SPR741 combined with partner antibiotics may result in improved potency and a broader spectrum of activity than that of the partner antibiotic alone. *In vitro* and *in vivo* studies showed that SPR741 combined with ceftazidime, piperacillin-tazobactam, and other antibiotics enhances the microbiological activity of the partner antibiotics against a wide range of Gram-negative pathogens including MDR strains (11–17) and anaerobic pathogens (18).

We report results from first-in-human studies with SPR741 monotherapy which examined the safety, tolerability, and pharmacokinetics (PK) after single and multiple ascending (SAD and MAD, respectively) intravenous (i.v.) doses (study SPR741-101s) as well as the effect of coadministration of SPR741 with partner antibiotics on the safety and PK profile of the individual components (study SPR741-102).

## RESULTS

### Subject disposition.

In the SAD phase, 64 subjects were randomized and analyzed for safety, and 48 provided PK data for SPR741. In the MAD phase, 32 subjects were randomized and analyzed for safety, and 24 provided PK data. In the drug-drug interaction (DDI) study, 27 subjects were randomized, received all study treatments, and completed the study with available PK data.

### Baseline characteristics.

For the SAD/MAD study, baseline demographic characteristics were well balanced between placebo and active treatment groups. In the SAD phase, mean (standard deviation [SD]) age ranged from 25.6 (6.0) to 28.1 (8.6) years across cohorts, 62 (97%) were male, 54 (84%) were white, and mean (SD) body weight was 76.8 (9.2) to 80.9 (10.1) kg. In the MAD phase, mean (SD) age was 28.0 (3.6) to 28.8 (5.8) years, all were male, 28 (88%) were white, and mean (SD) body weight was 75.1 (10.8) to 79.7 (8.9) kg. In the DDI study, all subjects were male, mean (SD) age was 36.7 (9.9) years, and mean (SD) body weight was 80.6 (8.5) kg. Twenty-six subjects were white, and one was Asian.

### Pharmacokinetics. (i) SAD/MAD study.

**FIG 1:**
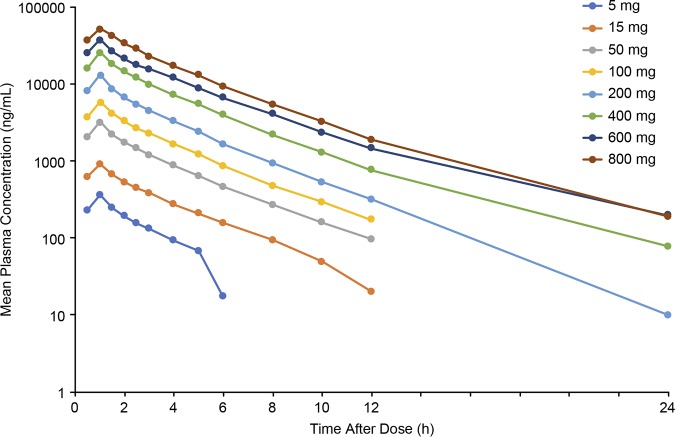
Mean plasma SPR741 concentrations (semi-log scale) in SAD phase (*n* = 6 per group).

Mean plasma concentrations of SPR741 peaked at approximately 1 h after single i.v. doses and declined over 24 h (Fig. 1). SPR741 displayed a linear and proportional PK profile when administered as single 1-h i.v. infusions at doses up to 800 mg to healthy subjects, with a mean half-life (*t*_1/2_) ranging from 2.0 to 3.8 h (Table 1).

**TABLE 1:** Mean of plasma PK parameters for SPR741 in the SAD phase (full PK population)

SPR741 dose (mg) (*n* = 6/dose)^*a*^	PK profile (CV%)^*b*^				
	*C*_max_ (ng/ml)	AUC_0-inf_ (h·ng/ml)	*t*_1/2_ (h)	Nominal dose	
				CL (liters/h)	*V* (liters)
5	370 (10.9)	1,010 (10.9)	2.02 (11.3)	5.00 (11.7)	14.4 (3.2)
15	924 (7.6)	3,092 (22.0)	2.81 (20.7)	5.04 (20.8)	19.7 (6.2)
50	3,252 (15.8)	9,745 (10.7)	2.48 (14.0)	5.18 (11.7)	18.4 (14.1)
100	6,008 (11.8)	18,151 (9.3)	2.57 (15.6)	5.55 (9.5)	20.6 (19.9)
200	13,367 (10.9)	37,304 (14.3)	2.62 (23.0)	5.46 (14.8)	20.3 (16.0)
400	26,017 (11.8)	82,232 (7.6)	3.47 (6.0)	4.89 (8.0)	24.5 (12.2)
600	38,050 (9.2)	133,110 (17.2)	3.76 (16.2)	4.62 (16.6)	24.6 (10.3)
800	52,617 (9.9)	189,706 (9.8)	3.46 (3.5)	4.25 (10.9)	21.2 (8.7)

a *n*, number of subjects.

b Arithmetic mean values are shown. For all doses, median *T*_max_ was 1.0 h. CV, coefficient of variation.

**FIG 2:**
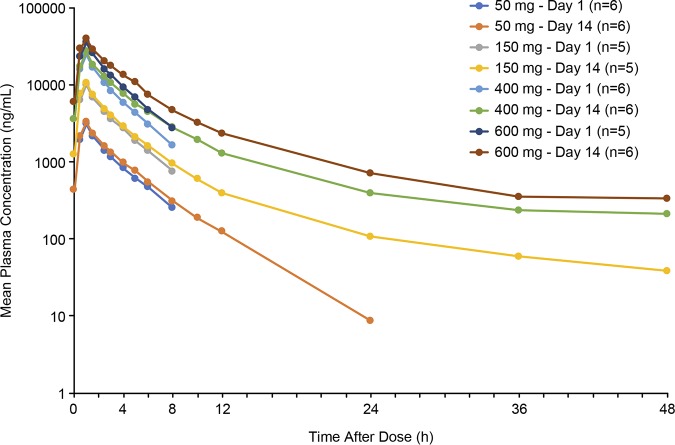
Mean plasma SPR741 concentrations (semi-log scale) in MAD phase.

**FIG 3:**
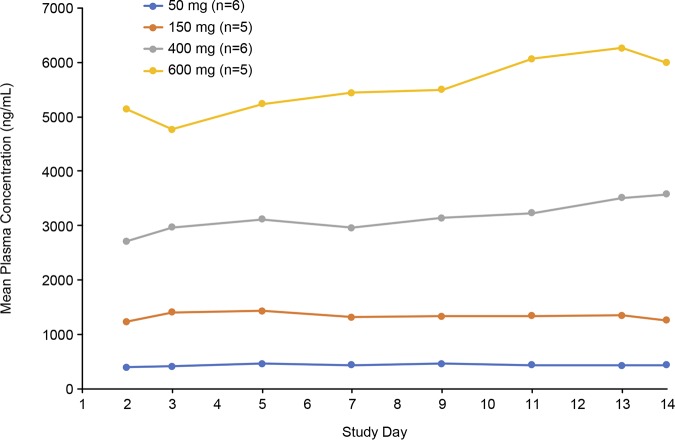
Mean trough plasma SPR741 concentrations in MAD phase.

When SPR741 was administered as a 1-h i.v. infusion of 50 to 400 mg every 8 h (q8h) for 14 days, plasma concentrations reached a peak level at approximately 1 h and declined over 48 h (Fig. 2); mean trough plasma concentrations remained stable from day 2 through day 14 (Fig. 3). The PK profile for SPR741 showed no evidence of accumulation or time-dependent changes in plasma exposure as measured by the area under the concentration-time curve from 0 to 8 h (AUC_0–8_) and the maximum concentration of drug in plasma (*C*_max_) (Table 2). Apparent terminal elimination half-life at day 1 was approximately 2.2 h, and at day 14 it ranged from 3.2 to 14.0 h.

**TABLE 2:** Mean of plasma PK parameters in the MAD phase (per-protocol PK analysis)

Day and SPR741 dose (mg [*n*])^*a*^	PK profile (CV%)^*b*^				
	*C*_max_ (ng/ml)	AUC_0–8_ (ng·h/ml)	*t*_1/2_ (h)	Nominal dose	
				CL (liters/h)	*V* (liters)
Day 1					
50 (6)	3,130 (10.8)	9,264 (15.5)	2.21 (14.5)	5.51 (16.0)	17.2 (8.9)
150 (5)	10,164 (14.2)	29,232 (17.7)	2.21 (14.8)	5.27 (18.7)	16.6 (15.3)
400 (6)	25,183 (20.8)	68,459 (11.3)	2.07 (7.6)	5.91 (11.5)	17.5 (4.0)
600 (5)	35,620 (16.6)	105,800 (17.0)	2.26 (15.8)	5.82 (18.8)	18.8 (17.7)
Day 14					
50 (6)	3,275 (14.9)	9,623 (10.2)	3.16 (48.8)	5.24 (10.1)	23.2 (38.8)
150 (5)	10,664 (16.9)	29,747 (19.3)	9.88 (61.9)	5.19 (18.4)	69.3 (44.8)
400 (6)	27,033 (19.9)	77,305 (20.8)	14.04 (52.6)	5.34 (18.7)	112.4 (67.8)
600 (5)	39,720 (13.6)	126,433 (10.6)	8.71 (17.4)	4.80 (12.2)	59.8 (15.1)

a *n*, number of subjects.

b Arithmetic mean values are shown. For all doses, median *T*_max_ was 1.0 h. CV, coefficient of variation.

**FIG 4:**
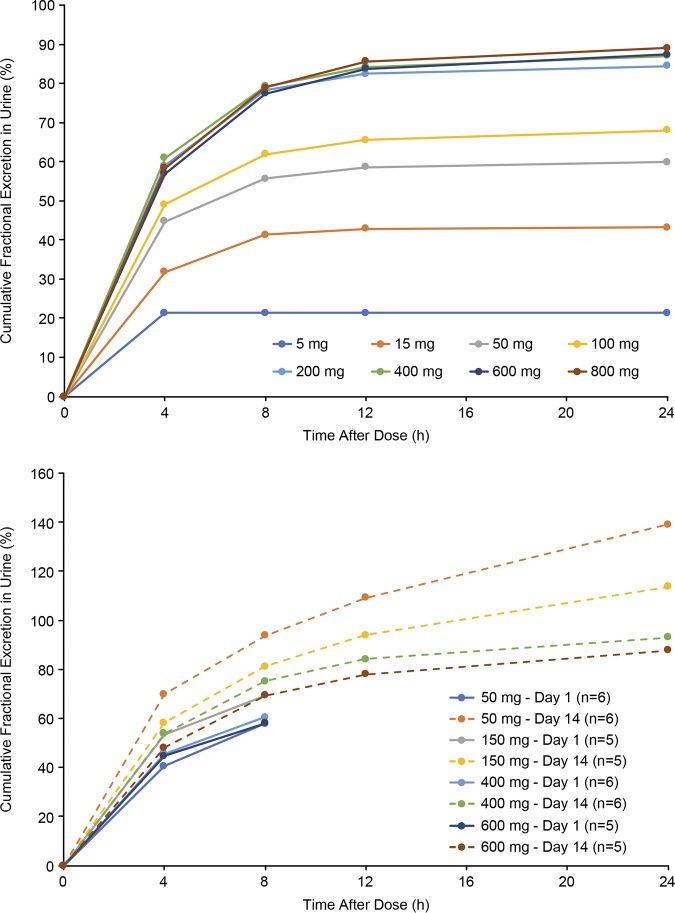
Cumulative fractional urinary excretion rates of SPR741 during the SAD phase (top) and the MAD phase (bottom).

In the SAD phase, the fraction of the SPR741 dose excreted in urine from 0 to 24 h ranged from 21.4% after a 5-mg dose to 89.0% after an 800-mg dose (Fig. 4). Renal clearance ranged from 1.23 (0.70) liters/h after a 5-mg dose to 3.8 (0.34) liter/h after an 800-mg dose.

In the MAD phase, fractional urinary excretion of SPR741 from 0 to 8 h on day 1 at doses of 50, 150, 400, and 600 mg was 57.9% to 69.4% of the dose (Fig. 4). The fraction of the dose excreted from 0 to 24 h postdose on day 14 ranged from 139.1% with 50 mg q8h to 87.8% with 600 mg q8h, suggesting saturation of renal elimination with increasing doses. Renal clearance from 0 to 8 h on day 1 ranged from 3.49 to 3.95 liters/h. On day 14, renal clearance from 0 to 24 h was 6.31 liters/h for a dose of 50 mg q8h, decreasing to 3.40 liters/h with a dose of 600 mg q8h, with the increase from day 1 to day 14 likely due to continued elimination from the previous dose. The decrease in renal clearance on day 14 with an increasing dose again suggests saturation of renal elimination at higher repeat doses.

### (ii) Drug-drug interaction study.

Simultaneous i.v. infusion of a single dose of 400 mg of SPR741 and any partner antibiotic (either 1.0 g of ceftazidime i.v., 4.5 g of piperacillin-tazobactam i.v., or 1.0 g of aztreonam i.v.) had no apparent effect on the concentration-versus-time profile of SPR741 in the presence of any of the partner antibiotics (Table 3). No evidence was noted of an increase or decrease in clearance or half-life of SPR741 in the presence of any of the partner antibiotics. The lack of any drug-drug interaction is supported statistically by comparable geometric least square mean (LSM) ratios for *C*_max_, the AUC from time zero to the last measurable time point (AUC_0-*t*_), and the AUC from time zero to infinity (AUC_0-inf_) (Table 4). All point estimates were within the 90% confidence interval (CI) for each parameter.

**TABLE 3:** PK parameters for SPR741 alone and combined with partner antibiotics and for ceftazidime, piperacillin, tazobactam, and aztreonam combined with SPR741 or administered alone

Drug and/or drug combination (*n*)^*a*^	Mean ± SD (CV [%])							
	*C*_max_ (ng/ml)	AUC_0-*t*_ (ng·h/ml)	AUC_0-inf_ (ng·h/ml)	*T*_max_ (h)	*k*_el_ (1/h)	*t*_1/2_ (h)	CL (liters/h)	*V* (liters)
SPR741								
SPR741 (27)	28,100 ± 3,030 (10.8)	89,700 ± 12,900 (14.4)	90,300 ± 13,000 (14.4)	1.0 ± 0.0 (0.6)	0.22 ± 0.04 (16.2)	3.2 ± 0.4 (12.0)	4.5 ± 0.8 (17.1)	20.6 ± 1.8 (8.8)
SPR741 + ceftazidime (9)	26,900 ± 1,720 (6.4)	85,500 ± 11,800 (13.8)	86,100 ± 11,900 (13.8)	1.0 ± 0.0 (0.0)	0.23 ± 0.05 (19.4)	3.1 ± 0.4 (14.3)	4.7 ± 0.6 (13.2)	20.9 ± 2.8 (13.4)
SPR741 + piperacillin-tazobactam (9)	27,100 ± 4,150 (15.3)	89,500 ± 16,800 (18.8)	90,000 ± 17,100 (19.0)	1.0 ± 0.0 (0.0)	0.21 ± 0.01 (6.2)	3.3 ± 0.2 (6.6)	4.6 ± 1.0 (22.5)	21.5 ± 4.2 (19.6)
SPR741 + aztreonam (9)	27,800 ± 3,090 (11.1)	95,300 ± 7,330 (7.7)	95,900 ± 7,420 (7.7)	1.0 ± 0.0 (4.4)	0.21 ± 0.01 (4.5)	3.4 ± 0.2 (4.5)	4.2 ± 0.4 (8.7)	20.4 ± 1.5 (7.4)
Ceftazidime								
Ceftazidime (9)	70,200 ± 5,790 (8.2)	162,000 ± 22,400 (13.8)	163,000 ± 22,500 (13.8)	1.0 ± 0 (0)	0.26 ± 0.01 (3.1)	2.7 ± 0.1 (3.1)	6.3 ± 0.8 (12.8)	24.0 ± 3.1 (12.8)
SPR741 + ceftazidime (9)	66,900 ± 8,140 (12.2)	162,000 ± 21,800 (13.5)	162,000 ± 21,900 (13.5)	1.0 ± 0 (0)	0.26 ± 0.01 (4.1)	2.6 ± 0.1 (4.1)	6.3 ± 0.8 (13.2)	23.8 ± 2.6 (10.9)
Piperacillin								
Piperacillin-tazobactam (9)	154,000 ± 23,500 (15.2)	240,000 ± 33,000 (13.7)	240,000 ± 33,000 (13.7)	1.0 ± 0 (1.2)	0.32 ± 0.02 (5.6)	2.2 ± 0.1 (5.7)	19.1 ± 3.0 (15.8)	60.7 ± 10.6 (17.4)
SPR741 + piperacillin-tazobactam (9)	155,000 ± 25,300 (16.3)	237,000 ± 31,900 (13.4)	238,000 ± 31,900 (13.4)	1.0 ± 0 (0)	0.33 ± 0.02 (6.7)	2.1 ± 0.1 (6.4)	19.3 ± 3.3 (16.9)	59.9 ± 11.9 (19.9)
Tazobactam								
Piperacillin-tazobactam (9)	18,500 ± 2,610 (14.1)	32,000 ± 5,060 (15.8)	32,000 ± 5,060 (15.8)	1.0 ± 0 (1.2)	0.40 ± 0.12 (30.8)	1.9 ± 0.4 (22.4)	16.1 ± 3.0 (18.4)	41.6 ± 6.5 (15.6)
SPR741 + piperacillin-tazobactam at (9)	18,300 ± 2,820 (15.4)	32,100 ± 5,020 (15.6)	32,100 ± 5,020 (15.6)	1.0 ± 0 (0)	0.41 ± 0.12 (29.5)	1.8 ± 0.4 (22.0)	16.0 ± 3.0 (18.5)	40.8 ± 11.3 (27.7)

a All drugs were administered i.v. Doses were as follows: SPR741, 400 mg; ceftazidime, 1.0 g; piperacillin-tazobactam, 4.5 g; aztreonam, 1.0 g. *n*, number of subjects.

**TABLE 4:**
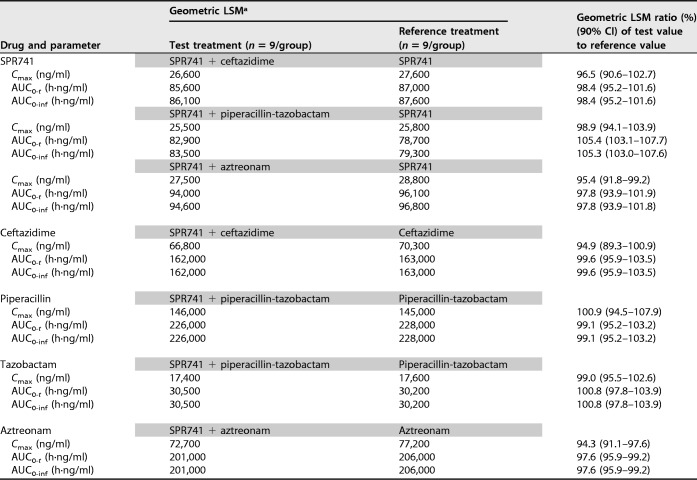
Summary of statistical analysis for SPR741 administered alone or combined with ceftazidime, piperacillin, tazobactam, and aztreonam

^a^All drugs were administered i.v. Doses were as follows: SPR741, 400 mg; ceftazidime, 1.0 g; piperacillin-tazobactam, 4.5 g; aztreonam, 1.0 g. *n*, number of subjects.

Simultaneous infusion of 1.0 g of ceftazidime i.v., 4.5 g of piperacillin-tazobactam i.v., or 1.0 g of aztreonam i.v. combined with 400 mg of SPR741 i.v. had no apparent effect on concentration-versus-time profiles of any of the partner antibiotics in the presence of SPR741 (Table 3). No increase or decrease in clearance or half-life was noted for any of the partner antibiotics in the presence of SPR741. The lack of any effect of coadministration on the PK profiles is supported statistically by comparable geometric LSM ratios for *C*_max_, AUC_0-_*_t_*, and AUC_0-inf_ (Table 4).

### Safety and tolerability. (i) SAD/MAD study.

SPR741 in single or multiple doses was well tolerated in the healthy adult subjects. In the SAD phase, 34 treatment-emergent adverse events (TEAEs) were reported for 15 of 48 (31%) SPR741-treated subjects and for 5 of 16 (31%) placebo-treated subjects; most events were mild in severity. No subject experienced a severe TEAE or serious adverse event (AE), and no deaths or discontinuations for TEAEs were reported. The most common TEAE in the SPR741 group was headache in 4 (8%) subjects (all unrelated to study drug); no other individual TEAEs were reported in more than 3 subjects. No subjects experienced a TEAE classified as a renal or urinary disorder, and no subject had an abnormal serum creatinine value.

In the MAD phase, at least one AE occurred in all subjects, and the most common systemic TEAEs were headache in 8 (33%) subjects, contact dermatitis in 7 (29%) subjects, decreased creatinine clearance (CrCl) in 6 (25%) subjects, and diarrhea in 3 (13%) subjects (Table 5). Only three TEAEs of moderate severity deemed related to study drug were reported in the MAD part of the study, two of which occurred in the placebo arm (tachycardia and staphylococcal bacteremia in the placebo arm and decreased creatinine renal clearance in the 600-mg SPR741 group). One subject in the SPR741 150-mg group with a history of electrocardiogram (ECG) abnormalities was withdrawn for a serious AE of atrial fibrillation that was determined to be probably related to treatment. Six (25%) subjects had decreases in CrCl with SPR741. For 5 of the 6 subjects, the events were mild in severity, serum creatinine remained within normal limits throughout dosing, and CrCl was normal at follow-up. One subject in the 600-mg group had a TEAE of decreased CrCl of moderate severity. Serum creatinine increased from 1.1 mg/dl at baseline to 1.79 mg/dl at day 16, which was associated with a 50% decline in CrCl from 110 ml/min to 65 ml/min. No deaths or severe TEAEs occurred. Catheter site pain (25%) and erythema (13%) related to the PK cannula occurred with SPR741. A total of 81 infusion site reactions (site bruising, erythema, pain, phlebitis, and swelling) related to i.v. drug infusion occurred with SPR741. Diarrhea occurred in 3 (13%) patients with SPR741. All infusion site reactions were mild, and no infusion site reactions led to premature discontinuation of study drug.

**TABLE 5:** Incidence of systemic TEAEs occurring in >1 subject with SPR741 in the MAD phase

TEAE type	Incidence by SPR741 dose (*n* = 6/group)^*a*^								Incidence with:			
	50 mg		150 mg		400 mg		600 mg		Active treatment (*n* = 24)		Placebo (*n* = 8)	
	No. (%) of subjects	No. of events	No. (%) of subjects	No. of events	No. (%) of subjects	No. of events	No. (%) of subjects	No. of events	No. (%) of subjects	No. of events	No. (%) of subjects	No. of events
All	6 (100)	23	6 (100)	45	6 (100)	42	6 (100)	68	24 (100)	178	8 (100)	63
Abdominal pain	1 (17)	1			1 (17)	1			2 (8)	2		
Arthralgia			1 (17)	1			1 (17)	2	2 (8)	3		
Back pain	2 (33)	2							2 (8)	2	1 (13)	1
Chest discomfort					1 (17)	1	1 (17)	1	2 (8)	2		
Contusion					1 (17)	1	1 (17)	1	2 (8)	2		
Creatinine clearance decrease	1 (17)	1	1 (17)	1	1 (17)	1	3 (50)	3	6 (25)	6		
Dermatitis contact	2 (33)	2	2 (33)	2	1 (17)	1	2 (33)	2	7 (29)	7	4 (50)	5
Diarrhea	1 (17)	1			1 (17)	1	1 (17)	1	3 (13)	3		
Dysesthesia	1 (17)	1	1 (17)	1					2 (8)	2		
Headache	1 (17)	1	4 (67)	4	1 (17)	1	2 (33)	2	8 (33)	8	3 (38)	4
Nausea			1 (17)	1			1 (17)	1	2 (8)	2	2 (25)	3
Rhinitis							2 (33)	2	2 (8)	2	1 (13)	1

a *n*, number of subjects.

### Drug-drug interaction study.

Twelve TEAEs occurred across all treatment groups, and all were of mild or moderate severity. Headache (4) and epistaxis (2) were the only adverse events occurring in more than one subject. No serious AEs or discontinuation for AEs was reported. No clinically significant changes were observed in clinical laboratory testing, vital signs, physical examination, or 12-lead ECG parameters. No clinically significant changes in blood urea nitrogen, serum creatinine, or creatinine clearance were observed in any treatment group.

## DISCUSSION

In the first-in-human SAD/MAD clinical study, SPR741 exhibited a linear and proportional PK profile when administered as a single i.v. infusion at doses up to 800 mg. In the MAD phase administering SPR741 doses of 50 to 400 mg q8h for 14 days, the plasma PK profile showed no evidence of accumulation or time-dependent changes in plasma exposure (AUC_0–8_, *C*_max_, or trough concentration [*C*_trough_]) although the half-life increased at day 14 compared with that at day 1. SPR741 was primarily excreted via the kidney, with 80% or more of the total dose recovered in the urine by 24 h by day 14.

Clinically significant DDI may occur with the administration of many antibiotics, including rifampin, macrolides, moxifloxacin, and linezolid (19, 20). Therefore, it was important to exclude any DDI potential when SPR741 was administered with partner antibiotics. No significant change in *C*_max_, AUC_0-_*_t_*, or AUC_0-inf_ for SPR741 or partner antibiotics occurred with simultaneous administration of single i.v. doses of SPR741 alone and in combination with ceftazidime, piperacillin-tazobactam, or aztreonam.

In general, SPR741 was well tolerated in both studies, and most study drug-related TEAEs were mild. While elevations in creatinine clearance were observed with SPR741 in 6 subjects in the MAD study, only 1 subject had a moderate increase in serum creatinine above baseline that began at day 14 of the 600-mg q8h dosage regimen and had resolved at follow-up by day 38. The remaining subjects had normal serum creatinine levels at a day 16 postdose evaluation. Although catheter site reactions were common in both the SAD and MAD parts of the study, these catheters were used only for obtaining PK and safety blood samples, and, as expected, none of the reactions were deemed related to study treatment. A greater number of subjects reported infusion site reactions in the MAD part of the study than in the SAD part. Infusion site phlebitis and thrombosis were uncommon (3 and 2 SPR741 subjects, respectively); all infusion site reactions were mild, and no infusion site reactions led to premature discontinuation of study drug.

Summaries of TEAEs by severity and relationship showed no apparent differences between treatments (SPR741 or placebo) and no dose-related trends in subjects who received SPR741 in either the SAD or MAD parts of the study.

In both the SAD and MAD parts of the study, there were no clear differences in safety as assessed by vital signs and ECG assessments in subjects who had received either single or multiple doses of SPR741 compared with those who received placebo, and there were no dose-related trends in these safety assessments in those subjects who received SPR741.

No renal-associated TEAEs occurred in any subject in the SAD part of the study; however, 6 TEAEs of CrCl decrease occurred in six SPR741 subjects in the MAD part, half of which occurred in the highest-dose cohort (600 mg i.v. q8h for 14 days). All decreases in CrCl were reversible and mild in severity except one moderately severe TEAE in a cohort 12 (600 mg) subject (CrCl decreased from 110 ml/min at baseline to 65 ml/min on day 16). A decrease in CrCl is a known effect of the polymyxin antibiotic class.

A need exists to expand the clinical utility of widely used antibiotic agents in high-risk patients by addressing the loss of potency of such drugs against Gram-negative strains of bacteria (7). The limitations of current antibiotics for treating serious infections has surfaced with the emergence of MDR strains of Escherichia coli, Klebsiella pneumoniae, and Actinobacteria baumannii. Infections due to MDR bacteria result in greatly increased morbidity and mortality and substantially increased economic costs (7, 21). While most MDR Gram-negative pathogens demonstrate some susceptibility to second- and third-line antibiotics, their use is limited by toxicity, cost of therapy, and efforts to restrict their use (3, 4).

The polymyxin analog SPR741 lacks significant direct antibacterial activity against Gram-negative organisms (MIC of 16 μg/ml for most species); however, when combined with antibiotics that have limited or no antibacterial activity against Gram-negative pathogens, SPR741 expands the spectrum of the partner antibiotic to include Gram-negative species (11, 12). SPR741 demonstrated the potential to lower MICs when combined with partner antibiotics, including bacterial strains that were beyond the susceptible breakpoint of the partner antibiotic (11, 12).

SPR741 demonstrates utility as a potentiator for use in combination with existing antibiotics for a wide variety of life-threatening infections caused by Gram-negative pathogens. The results from the SAD/MAD and drug interaction studies inform decisions on further development of SPR741 and potential partner antibiotics for combination use. Clinical studies are planned to explore the efficacy and tolerability of SPR741 combinations for treating serious infections due to MDR pathogens.

## MATERIALS AND METHODS

These studies were conducted according to the principles of the Declaration of Helsinki and Guidance on Good Clinical Practice. The study protocols, amendments, and informed consent forms were reviewed and approved by an Institutional Review Board. All subjects provided written informed consent prior to participating in any study activities.

### Study design. (i) SAD/MAD study (ClinicalTrials.gov identifier NCT03022175).

The SAD/MAD study was a phase 1, double-blind, placebo-controlled, multicohort trial. Part 1 used a SAD design according to which subjects received a single i.v. dose of SPR741. Part 2 used a MAD design in which subjects received multiple daily i.v. doses of SPR741 over a period of 14 days. The objective was to assess the safety/tolerability and PK of single and multiple ascending doses of SPR741.

In the SAD phase, healthy volunteers were screened within 28 days prior to dosing. Subjects were admitted to the clinical facility on day −1 for up to 3 days. A single dose of SPR741 (or placebo) was administered on day 1. Following completion of all safety assessments and sampling for PK analyses, subjects were discharged on day 2. A follow-up visit occurred 5 to 7 days after day 1 dosing. In the MAD phase, all subjects were admitted to the clinical facility on day −1. Dosing commenced on the morning of day 1. Three doses were administered daily at approximately 8 (±0.5)-h intervals for a total of 14 consecutive days. The last dose was administered on the morning of day 14. Blood and urine samples were collected for assessment of PK parameters. Subjects were discharged on day 16 following completion of all PK sample collection and safety assessments, and a follow-up visit occurred 5 to 7 days after the last dose.

### (ii) Drug-drug interaction study (ClinicalTrials.gov identifier NCT03376529).

The drug-drug interaction study was a phase 1, single-center, multiarm, open-label, randomized, three-period crossover study. The objective was to evaluate the DDI potential and the PK of SPR741 coadministered with each of three partner antibiotics as well as the DDI and PK effects of three partner antibiotics administered alone and with SPR741. The study comprised a screening phase (day −28 to day −2), three treatment arms (day 1 to day 8 for each arm), and an end-of-study follow-up (day 9). Each subject participated in screening, one treatment arm, and the end-of-study follow-up. The maximum duration of participation for each subject was approximately 37 days. Subjects remained in the clinical unit from the day before dosing (day −1) to 24 h after the third and last dose period (day 8).

### Subject selection.

Healthy adult subjects aged 18 to 55 years with a body mass index of 18.5 to 29.9 kg/m^2^ and weight between 62.5 and 100 kg (SAD/MAD) or 55 and 100 kg (DDI) were eligible. Subjects were medically healthy with no clinically significant abnormalities based on physical examination, vital signs (temperature, heart rate, blood pressure, and respiratory rate), ECG, and clinical laboratory testing (serum chemistry, hematology, and urinalysis). All subjects were nonsmokers, females were of non-childbearing potential, and males used an acceptable form of contraception. Subjects were excluded for any clinically significant medical condition, a history of Clostridium difficile infection, positive human immunodeficiency virus (HIV) antibody, hepatitis B surface antigen (HBsAg), or hepatitis C antibody, positive urine drug/alcohol test or history of substance or alcohol abuse, documented hypersensitivity or anaphylaxis to any medication, or use of any prescription or over-the-counter medications with 7 days of randomization.

### Study treatments. (i) SAD/MAD study.

In the SAD phase, subjects were randomized to one of eight cohorts that received doses of 5, 15, 50, 100, 200, 400, 600 or 800 mg of SPR741. Within each cohort, two subjects received placebo, and six received SPR741. In cohort 1, two subjects (sentinels) were dosed with SPR741 or placebo 48 h prior to dosing of the remaining subjects. The remaining six subjects were dosed only after no safety concerns were identified in the sentinel subjects. After each dose cohort had completed administration of study drug and evaluation, a Safety Monitoring Group reviewed blinded cumulative safety data (including day 5 to day 7 follow-up data) to confirm the safety and tolerability of SPR741.

The MAD phase of the study began after safety and tolerability were confirmed in cohort 5, and the appropriate dose level was established. Two subjects received placebo, and six subjects received SPR741 doses of 50, 150, 400 or 600 mg q8h for 14 days. In the first cohort of the MAD phase (50 mg q8h), two subjects (sentinels) began dosing with SPR741 or placebo 72 h prior to dosing of the remaining subjects in this cohort. The remaining six subjects were dosed only after no safety concerns were identified by the Safety Monitoring Group at 72 h. After each MAD dose cohort had completed administration of study drug and all evaluations, the Safety Monitoring Group reviewed blinded cumulative safety data (including the day 19 to 21 follow-up data) to confirm the safety and tolerability of study drug.

### (ii) Drug-drug interaction study.

The DDI study consisted of three treatment arms with three dosing periods in each arm (days 1, 4, and 7). In random sequence, subjects received a single dose of study treatments with a 2-day washout period between each assigned treatment. In treatment arm 1, subjects received 400 mg of SPR741 i.v. over 1 h, 400 mg of SPR741 i.v. over 1 h plus 1.0 g of ceftazidime i.v. over 1 h, and 1.0 g of ceftazidime i.v. over 1 h. In treatment arm 2, subjects received 400 mg of SPR741 i.v. over 1 h, 400 mg of SPR741 i.v. over 1 h plus 4.5 g of piperacillin-tazobactam i.v. over 1 h, and 4.5 g of piperacillin-tazobactam i.v. over 1 h. In treatment arm 3, subjects received 400 mg of SPR741 i.v. over 1 h, 400 mg of SPR741 i.v. over 1 h plus 1.0 g of aztreonam i.v. over 1 h, and 1.0 g of aztreonam i.v. over 1 h.

### Study assessments.

For both studies, safety assessments included clinical laboratory testing (hematology, coagulation, serum chemistry, renal chemistry, and urinalysis), vital signs (blood pressure, heart rate, body temperature, and respiratory rate), physical examination, and triplicate 12-lead ECGs. In the MAD phase, changes in 24-h creatinine clearance (CrCl) levels based on plasma and urine creatinine concentrations were determined at baseline and following the last dose of study drug on day 14. Adverse events were recorded at each study visit.

### Pharmacokinetic analysis.

For both studies, maximum plasma concentration (*C*_max_), area under the concentration-time curve from time zero to the last measurable time point (AUC_0-t_), area under the concentration-time curve from time zero to infinity (AUC_0-inf_), time to maximum concentration (*T*_max_), terminal elimination rate constant (*k*_el_), terminal half-life (*t*_1/2_), terminal clearance (CL), and volume of distribution (*V*) were determined. PK parameters were determined using WinNonlin Phoenix, version 6.4.

In addition, the area under the concentration-time curve from 0 to 24 h after the start of infusion (AUC_0–24_) was determined for the SAD phase, and the areas under the concentration-time curves from time zero to 8 h from start of infusion (AUC_0–8_) on day 1 and from 0 to 48 h (AUC_0–48_) following the last dose on day 14 as well as predose trough concentrations on days 2, 3, 5, 7, 9, 11, and 13 were determined for the MAD phase.

For the SAD phase, blood samples were obtained predose (within 10 min), at 30 min following the start of infusion, at the end of infusion, and at 75, 90, 105, 120, and 150 min and 3, 4, 5, 6, 8, 10, 12, and 24 h following the start of infusion. For the MAD phase, blood was obtained predose (within 10 min), at 30 min following the start of infusion, at the end of infusion, and at 90 and 150 min and 3, 4, 5, 6, and 8 h following the start of infusion (day 1, first dose; day 14, last dose). Five additional blood samples were obtained at 10, 12, 24, 36, and 48 h following the start of infusion of the last dose (morning of day 14). For the DDI study, blood samples for determining plasma concentrations of SPR741 and each partner antibiotic were obtained predose (within −10 min), at 30 min, and at 1 h, 1 h 15 min, 1 h 30 min, 1 h 45 min, 2, 4, 8, 12, and 24 h postdose for each dose period. Plasma samples for drug concentration measurements were analyzed using a validated high-pressure liquid chromatography tandem mass spectrometry (HPLC-MS/MS) method. In brief, samples were spiked with a stable isotope-labeled internal standard and then extracted by protein precipitation before injection onto the HPLC-MS/MS system. Unique transitions were monitored for all test articles and internal standards. Plasma concentrations below the limit of quantitation were reported as 0.

### Statistical analysis.

Plasma concentrations and PK parameters for SPR741, ceftazidime, piperacillin, tazobactam, and aztreonam were summarized for each treatment using descriptive statistics. In the SAD/MAD study, geometric means were calculated for AUC and *C*_max_ values. Analyses using linear models were performed to assess dose proportionality (both single dose and multiple dose), time dependence, accumulation, and attainment of steady state (multiple dose). Dose proportionality was assessed using linear models for the SAD and MAD cohorts separately. Dose linearity of *C*_max_ values and AUCs across the dose range were assessed by fitting the following power model and testing for β = 1 using a generalized linear model. In the DDI study, log-transformed *C*_max_, AUC_0-_*_t_*, and AUC_0-inf_ for SPR741, ceftazidime, piperacillin, tazobactam, and aztreonam were compared with analysis of variance (ANOVA) including fixed effects for treatment, period, sequence, and subject nested within treatment sequence. Point estimates and 90% confidence intervals (CIs), using the residual mean square error obtained from the ANOVA, were constructed for the comparisons between treatments. The point and CI estimates were back-transformed to give estimates of the ratios (percentages) of the geometric LSMs and corresponding 90% CIs.
